# Tissue Engineering to Repair Diaphragmatic Defect in a Rat Model

**DOI:** 10.1155/2017/1764523

**Published:** 2017-08-27

**Authors:** G. P. Liao, Y. Choi, K. Vojnits, H. Xue, K. Aroom, F. Meng, H. Y. Pan, R. A. Hetz, C. J. Corkins, T. G. Hughes, F. Triolo, A. Johnson, Kenneth J. Moise, K. P. Lally, C. S. Cox, Y. Li

**Affiliations:** ^1^Department of Pediatric Surgery, University of Texas, McGovern Medical School at Houston, Houston, TX, USA; ^2^Center for Stem Cell and Regenerative Medicine, University of Texas Health Science Center at Houston (UT Health), Houston, TX 77030, USA; ^3^Department of Orthopaedic Surgery, University of Texas, McGovern Medical School at Houston, Houston, TX, USA; ^4^Department of Obstetrics, Gynecology, and Reproductive Sciences, University of Texas, McGovern Medical School at Houston, Houston, TX 77030, USA

## Abstract

Tissue engineering is an emerging strategy for repairing damaged tissues or organs. The current study explored using decellularized rat diaphragm scaffolds combined with human amniotic fluid-derived multipotent stromal cells (hAFMSC) to provide a scaffold, stem cell construct that would allow structural barrier function during tissue ingrowth/regeneration. We created an innovative cell infusion system that allowed hAFMSC to embed into scaffolds and then implanted the composite tissues into rats with surgically created left-sided diaphragmatic defects. Control rats received decellularized diaphragm scaffolds alone. We found that the composite tissues that combined hAFMSCs demonstrated improved physiological function as well as the muscular-tendon structure, compared with the native contralateral hemidiaphragm of the same rat. Our results indicate that the decellularized diaphragm scaffolds are a potential support material for diaphragmatic hernia repair and the composite grafts with hAFMSC are able to accelerate the functional recovery of diaphragmatic hernia.

## 1. Introduction

Tissue engineering technologies have developed rapidly in the last decade. Several successful bioengineered tissues are undergoing evaluation in clinical trials. Recently, decellularized tissue has been used as a scaffold to grow organs, including a functional heart, lung, intestines, and other organs [1, 2]. The process of decellularization can remove resident cells from the donor organs or tissues using detergent and mechanical agitation, leaving a three-dimensional (3D) extracellular matrix (ECM) scaffold that can be reseeded with new progenitor cells or composites [3–5]. The benefits of decellularized ECM scaffolds include preservation of the natural organ architecture as well as maintenance of microvascular networks [4, 6, 7]. Thus, decellularized scaffolds have gained popularity and are becoming a common scaffold for whole organ regeneration. In larger organs with intact macrovasculature, recellularization with stem cells can be accomplished by intravascular infusion. In smaller tissues, this is more difficult. To overcome this in our model, we developed a perfusion apparatus to allow pressurized donor cell infusion into an ECM scaffold.

Congenital diaphragmatic hernia (CDH) is a congenital diaphragmatic defect associated with pulmonary hypertension and cardiopulmonary failure that continues to present a challenge for neonatologists and pediatric surgeons [8–10]. While the incidence of CDH varies between 1 : 2000–4000 live births, the hospital costs exceeds 100 times the cost of an uncomplicated birth [11]. Small defects described as types A and B by the Congenital Diaphragmatic Hernia Study Group can be repaired primarily [12]. However, larger types C and D defects require patch repair [13]. Although the early mortality associated with CDH has decreased to 5–10% due to improved neonatal intensive care, the long-term morbidity associated with patch repairs remains significant, including musculoskeletal chest wall deformities (67%), scoliosis (13%) as well as small bowel obstruction (13%), and failure to thrive (78%) with many infants at less than 50% percentile in weight at 24 months post discharge [14, 15]. In the last decade, clinical and preclinical investigators have been investigating the use of biological patches for CDH repair and have included lyophilized dura, small intestine submucosa (SIS), and acellular dermis (Alloderm®) [16–18]. Biological patches alone have failed due to lack of tissue ingrowth with subsequent resorption of the patches, poor immediate strength, rupture, neighbor tissue adhesions, and fibrosis. Tissue-engineered patches for CDH repair seek to improve biomechanical compatibility while reducing recurrent hernia [18–23]. Decellularized ECM scaffolds have the potential of regenerating the structure and function of their native tissue over commercially available matrices from other tissues. Those decellularized ECM scaffolds have been used in combination with stem cells to construct composite tissues that have been utilized successfully in tissue repair, including diaphragmatic repair [23–25]. While the current practice of PTFE/Gore-tex® patch repair is reasonably effective with acceptable rates of recurrence and infection, a simple biologic tissue could represent an advantage, especially on the large diaphragmatic defects.

In the current study, we explored using a biological patch comprised of decellularized ECM scaffolds from rat diaphragms seeded with human amniotic fluid-derived multipotent stromal cells (hAFMSC), to repair a surgical diaphragmatic defect in a rat model. Structural and functional measures were used to define treatment outcome. We aimed to test whether a decellularized ECM scaffold recellularized with amniotic-derived stem cells can construct a functional composite tissue for diaphragmatic defect repair in a rat model.

## 2. Materials and Methods

### 2.1. Decellularized ECM Scaffolds from Rat Diaphragms

The procedure of tissue decellularization is to effectively remove cellular components and residual DNA, but keep the physicochemical structure of the ECM to support seeding cells' survival in a 3D architecture [25, 26]. Our protocol includes (1) excision of the rat hemidiaphragm in a sterile environment; (2) put the diaphragm in a tube with 40 mL PBS (50 mL, BD); (3) transfer the diaphragm into a 50 mL tube prefilled with 0.5% SDS; (4) attach tube to rocker (Nutator, number 421105, Beckton Dickinson, MD) and rotate for 0.5/sec for 24 hours; (5) transfer the diaphragm to a fresh tube prefilled with 0.5% sodium dodecyl sulfate (SDS) and rotate for 24 hours; (6) transfer the decellularized tissue into a 50 mL tube with sterile PBS and wash during rotation for 24 hours; and (7) leave the tissue in PBS and store at 4°C.

### 2.2. Mechanical Testing for Rat Diaphragm or Decellularized ECM Scaffolds

Six Sprague Dawley (SD) adult rats (female, 150–175 grams, approximately 7-8 weeks old, Charles River Lab.) were used to biotype donor diaphragms. After successful decellularization, the rat diaphragm ECM tissues were prepared as 5 mm wide × 10 mm long strips and mounted in a physiological perfusion chamber, interfaced to a force transducer that measures contractile force via a multitier Power Lab monitor (Experimetria Ltd. Balatonfured, Hungary). Once the tissue is mounted on the transducer, the hook and thread connected to the transducer are pulled taut so any subsequent contractions pull the force transducer down, displacing the sensor. This displacement, enhanced by the amplifier, is then graphically displayed on the computer screen versus time. Maximum force readings of ~6.0 grams were set for these measurements, since over 6.0 tears the ECM scaffolds.

### 2.3. Transmission Electron Microscopy (TEM) Scans for Decellularized ECM Scaffolds

Samples of ECM scaffolds were fixed with a TEM preparing solution containing 3% glutaraldehyde plus 2% paraformaldehyde in 0.1 M cacodylate buffer (pH 7.3), for 1 hour at ambient temperature, and then washed with cacodylate buffer (0.1 M, pH 7.3) for 3 × 10 minutes. After fixation, the samples were sequentially treated with Millipore-filtered 1% aqueous tannic acid for 30 minutes and 1% aqueous uranyl acetate for 1 hour in the dark. Then, the samples were dehydrated with a series of increasing concentrations of ethanol for 5 minutes each and then transferred to a series of increasing concentrations of hexamethyldisilazane (HMDS, 5 minutes each) and air-dried overnight. Samples were mounted on to double-stick carbon tabs (Ted Pella, Inc., Redding, CA), which had been previously mounted on to aluminum specimen mounts (Electron Microscopy Sciences, Ft. Washington, PA), and then coated under vacuum using a Balzer MED 010 evaporator (Technotrade International, Manchester, NH) with platinum alloy for a thickness of 25 nm and then immediately flash carbon coated under vacuum. The samples were transferred to a desiccator for examination at a later date. All samples were read in a JSM-5910 scanning electron microscope (JEOL, USA, Inc., Peabody, MA) at an accelerating voltage of 5 kV.

### 2.4. hAFMSC Isolation and Identification

hAFMSCs were isolated via amniocentesis (2–5 mL) from pregnant women (18 years and older, at least 16 weeks 0/7 days pregnant in sterile fashion during routine diagnostic amniocenteses from patients at the Texas Center for Maternal and Fetal Treatment (Institutional Review Board HSC-MS-111-0593). These samples were stored at 5°C after collection and processed within 48 hours from procurement (Institutional Biosafety Committee). Sample processing, including the isolation and expansion, was carried out in an ISO7 human cell production facility, in compliance with current good manufacturing practice (cGMP) guidelines of the Food and Drug Administration (FDA). These cells were identified based on their surface markers by immunostaining with flow cytometry analysis. We selected one donor-derived hAFMSC for the current study (from a 33-year-old healthy Caucasian female, nonhispanic donor at gestational age 20 weeks). The selected hAFMSCs were prelabeled with green fluorescent protein (GFP) as a track marker by using a lentivirus vector (Invitrogen) according to the manufacturer's instruction.

Following expansion, the aliquots were stored in liquid nitrogen for further studies. To confirm immunophenotype and differentiation capacity, hAFMSCs were tested for hallmarks via cell surface markers: CD90, CD73, CD45, CD34, CD29, CD106, CD10, CD13, HLA-ABC, and HLA-DR by flow cytometry, consistent with the International Society for Cellular Therapy (ISCT) definition of mesenchymal stem cells (MSCs) cell surface markers. We also evaluated their multiple-differentiation abilities in vitro, including osteogenic, adipogenic, chondrogenic, and myogenic differentiation, as described by others and in our previous reports [27–31].

### 2.5. Design and Creation of an Infusion System for Cell Seeding

**Figure 1:**
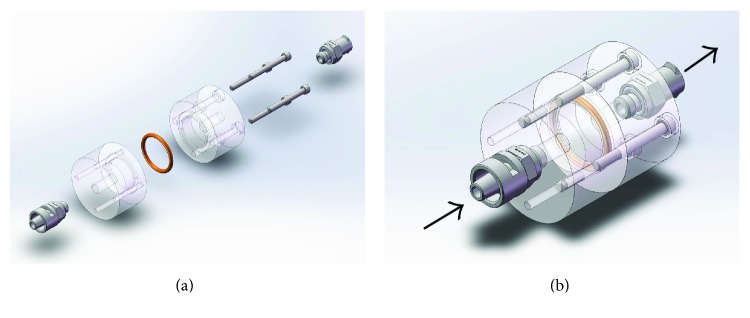
A scheme design of cellular infusion system. Two acrylic cylindrical paths allow pressurized perfusion of cell containing medium through a scaffold that results in cell seeding of the various layers of the scaffold. (a) Infusion system components. (b) Medium flow direction.

An infusion system was designed to create a uniform distribution of donor cells within a scaffold (Figure 1). The flow/medium influence is able to transport donor cells and distribute into the deep portion of decellularized ECMs. Briefly, donor diaphragm ECM scaffolds (adult female rats) were sandwiched in an infusion chamber, with media and cells pushed across the scaffold via hydrostatic pressure. The chamber consists of two pieces of polycarbonate, a sealing O-ring, luer connector fittings, and a set of machine screws to secure the polycarbonate pieces together. An exploded and assembled view of a CAD model of the device is shown in Figures 1(a) and 1(b), respectively. The polycarbonate pieces were milled from stock 1″ rod to create a small fluid chamber, a through-hole down the center, several through-holes circumferentially, and a small groove to allow for proper seating of the O-ring. The center through-holes were threaded to accept a luer fitting. The circumferential holes of one of the polycarbonate parts were also threaded to accept the clamping screws. After machining, the parts were vapor polished using dichloromethane to improve optical transparency of the device. For each ECM, we seeded it in 1 × 10^4^ fAFMSC (diluted in 1 mL PBS); subsequently, those composite tissues were transported (4°C) for surgery to repair the diaphragm defects created in the rat model.

### 2.6. Surgical Repair Diaphragmatic Defect in a Rat Model

All surgical procedures were approved by the Animal Welfare Committee (AWC) of the McGovern School of Medicine University of Texas Medical School at Houston (Protocol number AWC-12-080). SD rats (used number of rats: *n* = 40; 150–175 grams, 7 weeks old, female, Charles River Lab.) were anesthetized with 4% isoflurane and underwent endotracheal intubation under direct visualization. The animals were then placed on a heated operating platform and connected to a volume-controlled ventilator at a rate of 85 breaths per minute and tidal volume of 10 cc/kg with 2% maintenance isoflurane. After the appropriate anesthetic plane was confirmed by toe pinch, the rats were then sterilely prepared and draped to expose the upper thorax and abdomen. A 1.5 cm midline incision was created, beginning just inferior to the level of the xiphoid process. After entering the abdomen, a self-retaining abdominal wall retractor was used to expose the liver and diaphragm. A 3-0 chromic suture was then passed through the xiphoid and skin and secured in a sterile fashion to a fixed retractor to further expose the diaphragm.

**Figure 2:**
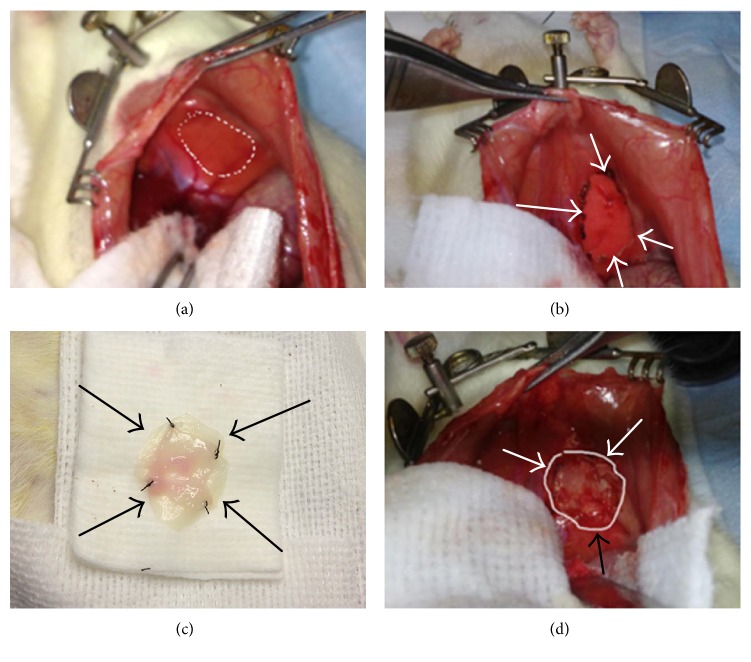
The surgical procedure to create the diaphragmatic defect and repair. (a) The rat diaphragm is explored through an abdominal approach, and the surgical defect is created. (b) A standardized defect size of 1.2 × 1.2 cm in the left side of the diaphragm is created. (c) A composite engineered tissue is prepared for implantation. (d) Complete repair of the defect by a composite engineered tissue.

Electrocautery was used to detach the falciform ligament, and a fined tooth forceps was used to grasp the center of the left hemidiaphragm, providing inferior gentle retraction away from the thoracic cavity; a small defect allowed insertion of the tip of the electrocautery device to breach the negative pressure of the thoracic cavity. An 18-gauge angiocather serving as a chest tube was then introduced into the thoracic cavity under direct visualization. A 1.2 × 1.2 cm^2^ circular diameter defect (about 75% tendon and 25% muscle-like tissues represent a total of ~40% of the total hemidiaphragm) was created in the left hemidiaphragm using electrocautery, and the experimental patch was sutured to the diaphragm in an underlay fashion with 2-3 mm overlap using 4-6 interrupted 5-0 Prolene@ sutures. The repair procedure is shown in Figures 2(a) and 2(b).

To close the abdomen, the pneumothorax was evacuated with a 10 cc syringe. Cefazolin (60 mg/kg) was administered directly to the peritoneum, and 0.25% bupivacaine infiltrated into the peritoneum and subcutaneous tissues along the incision. The incision was then closed with 4-0 Vicyrl@ sutures in two layers.

### 2.7. Biomechanical Stretch Testing

Currently, there are no methods that have been reported for testing diaphragmatic functions. We created a physiological test for rat diaphragmatic function based on our previous reports of skeletal muscle functional tests [32–34], via an electric stimulation to measure the rat diaphragm contractility ex vivo. At 4 and 6 months postsurgery, rats were anesthetized with 4% isoflurane and the chest and abdomen were exposed using a single midline incision. The sternum was bisected two rib levels above the diaphragm and carried posteriorly to the spine. After cardiac puncture, the segment of the dissected ribcage was then removed en bloc to include the entire diaphragm. The ribcage was trimmed so that the diaphragm was supported by just one rib level and placed in Kreb's solution. The diaphragm was then bisected to form two triangular segments, the right segment representing native diaphragm and the left representing the patch. Each triangle segment had a rib as the base and the central tendon at the apex. Both segments were then attached to a muscle stimulation platform to support a rib on one end. The segment was then passed through two metallic rings to create a uniform electric field. A 5-0 silk suture was then tied to the central tendon at the apex of the triangular segment. Each platform, containing one diaphragm segment was placed and secured to an organ bath containing Kreb's solution and connected to an oxygen supply. The sutures were then tied to a precalibrated cantilever for force transduction using LabChart ADInstruments software (Colorado Springs, CO). The diaphragm segments were prestretched to 1.0 gram and stimulated with 10 volts to generate a baseline force versus time curve. The diaphragm segments were then allowed to equilibrate for 30–45 minutes to allow for maximal recovery of force generation, and sequential stimuli are given with 1.0 gram prestretch; the contractile force was measured. The elasticity of the tissue segments was also measured by sequentially elongating the tissue segments by 1.0 mm increments and measuring both the passive or stimulated maximum force for each incremental stretch.

### 2.8. Histological Analysis

The repaired diaphragms were biopsied, and the biopsied sites were selected from the repaired edge parts, for histological analysis. Serial 8–10 microcryostat sections were performed for histological analyses. Hematoxylin and Eosin (H&E) staining identified the muscle tissue. Trichrome staining was used to analyze the collagen content (reflecting tendon-like tissue formation) of the repaired site tissues. The tissue slides were processed as specified in the manufacturer's protocol (Masson's Trichrome Stain Kit, K7228 IMEB, Inc.); thus, the nuclei were stained black, muscle fibers were stained red, and all collagen was stained blue. For immunohistochemistry, we used 10% horse serum (HS) to block nonspecific background binding and then applied rabbit anti-Nestin (1 : 100, Santa Cruz Biotechnology) and biotin-conjugated CD31 (1 : 150, Chemicon). Secondary fluorescent antibodies (1 : 500, anti-rabbit-594 and 1 : 800, Streptavidin-594) were applied, and then 4′,6-diamidino-2-phenylindole (DAPI, Sigma) was used to stain nuclei as described in our previous reports [35, 36]. Negative controls were performed concurrently with all immunohistochemical staining. Immunofluorescent results were visualized using fluorescent microscopy (Nikon microscope, Nikon, Melville, New York). We also captured fluorescent images from 3 to 5 different locations/slide by using the 20x objective and FITC or DSRED channels without recording DAPI. Then, images were analyzed using ImageJ software (NIH, USA), measuring the mean grey values of the threshold area. Data were collected in Excel for further analyses. The resultant values represented the mean of 3 experimental and 4 biological replicates.

### 2.9. Statistical Analysis

The statistical significance of differences between the groups of surgical treatment and control nonsurgery diaphragms was determined by using one-way analysis of variance (ANOVA) tests. Once finding a significance, for example, *p* values <0.05%, post hoc analysis was performed using Fisher's least significant difference (LSD) correction. Statistical significance was considered with a *p* < 0.05.

## 3. Results

### 3.1. Isolation and Characterization of Human Amniotic Fluid-Derived Multipotent Stromal Cells (hAFMSCs)

We obtained the human donor-derived hAFMSCs from a healthy 33-year-old Caucasian female, at gestational age 20 weeks. In brief, the human amniotic fluid was centrifuged at 400*g* for 15 minutes and the pellet was resuspended in complete TheraPEAK xeno-free mesenchymal stem cell growth medium (Lonza, Basel, CH) supplemented with 20% allogeneic pooled human AB serum (Valley Biomedical, Winchester, VA), 5 ng/mL basic fibroblast growth factor (CellGenix, Portsmouth, NH), and 50 *μ*g/mL gentamicin (Irvine Scientific, Santa Ana, CA) and the cells were cultured at 37°C in a 5% CO_2_ and 95% RH environment. The expansion was carried out under the same conditions changing the medium every 3–5 days. Upon reaching 80–90% confluence, cells were passaged by lifting with TrypLE Express xeno-free reagent (Thermo Fisher Scientific, Waltham, MA) and plating at a cell density of 900–1000 cells/cm^2^. Cells were cryopreserved in CryoStor CS10 medium (Biolife Solutions, Bothell, WA). Prior to transplantation, passage 3 cells were thawed, washed, and incubated in antibiotic-free medium and tested for mycoplasma, sterility (gram stain and 14 day aerobic and anaerobic cultures), endotoxin, and immunophenotype.

**Figure 3:**
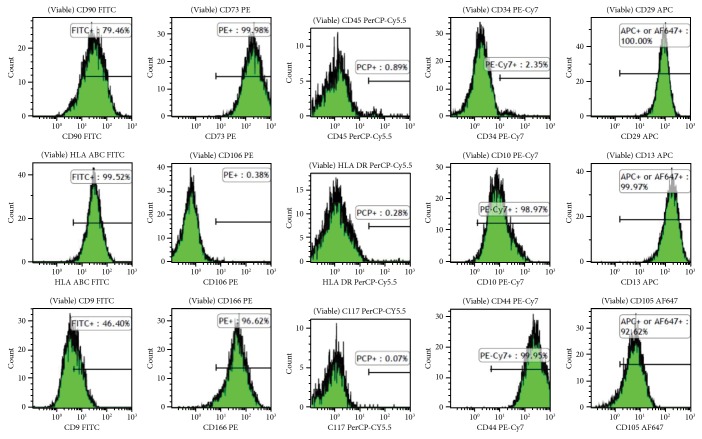
Flow cytometry analysis for hAFMSCs. hAFMSCs used for the current experiments that expressed surface markers as the percentage of viable singlets gated showed 100% CD29, 79.5% CD90, 0.9% CD45, 100% CD73, 2.4% CD34, 100% CD13, 99.5% HLA-ABC, 0.3% HLA-DR, 0.4% CD106, 99%, CD10, 92.6% CD105, 46.4% CD9, 0.1% CD117, 96.6% CD166, and 99.9% CD44.

**Figure 4:**
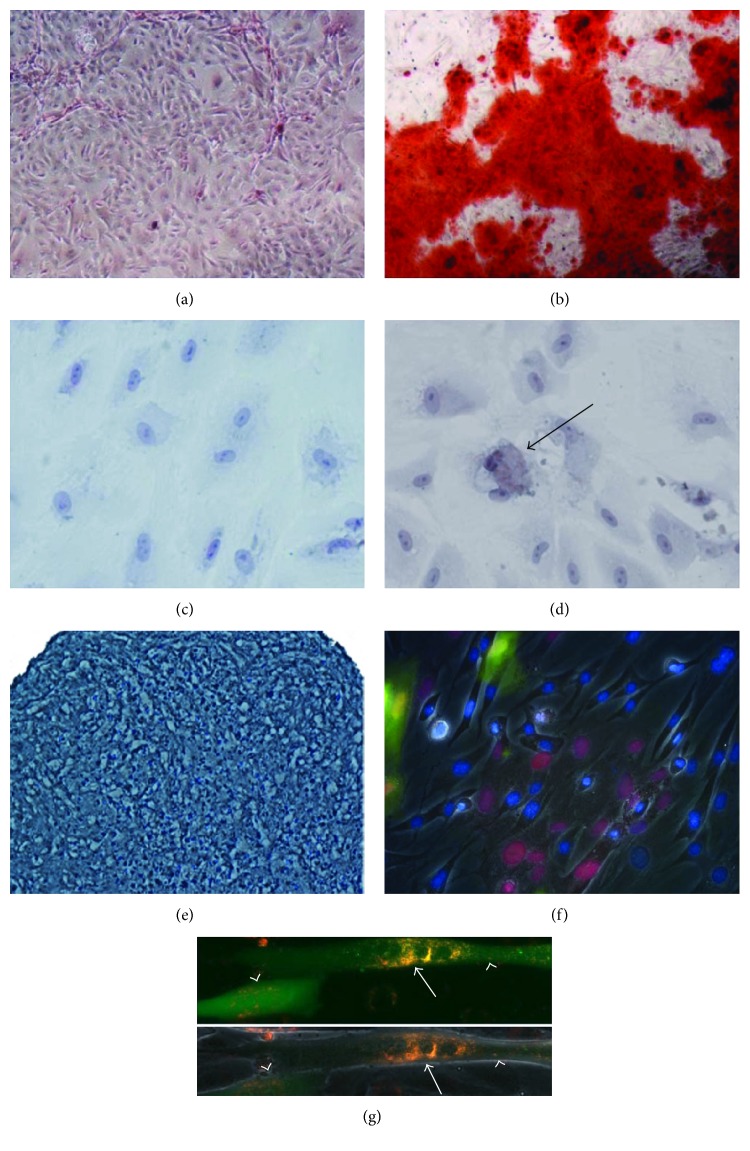
Multiple-differentiation potentials of hAFMSCs in vitro. Osteogenic differentiation of hAFMSC induction for 5 days, stained with Alizarin red: (a) noninduced control hAFMSCs and (b) induced hAFMSCs cultured in osteogenic medium containing BMP4. Adipogenesis of hAFMSCs after induction: (c) noninduced control hAFMSCs and (d) induced hAFMSCs within adipogenic medium for 5 days, as shown by positive Oil red O (arrows' cells). (e) Chondrogenic differentiation of hAFMSCs within chondrogenesis medium with Toluidine blue staining in vitro. Using a coculture system, the same number of both myoblasts (arrows, C2C12-prelabeled red dye-bits) and hAFMSCs (arrowheads, pre-GFP-labeled, green) were 1 : 1 mixed (f), per placing on to one dish. The fused multinucleated myotubes could be detected at 4 days after coculturing, in which some of the myotubes were positive for both red dye and green-GFP together (g). Results suggested that the myogenic differentiation potential via the myotube-fusion occurred between myoblasts and hAFMSCs.

These human donor-derived hAFMSCs were subsequently identified based on their surface markers through flow cytometry analysis, as in our previous reports [27, 29]. These cells were released for infusion upon meeting specific release criteria, including the following immunophenotype: <5% CD34+, CD45+, CD117, HLA-DR cells; >90% CD29+, CD73+, CD44+, CD13, CD166 cells, negative mycoplasma, and gram stain. The immunophenotyping results of the lots used in this work are shown in Figure 3. Those hAFMSCs also demonstrated tri-lineage differentiation potential, including osteogenic, adipogenic, chondrogenic, and myogenic differentiation (Figure 4) [27, 29, 37].

### 3.2. Tissue Decellularization and Cellular Infusion In Vitro

**Figure 5:**
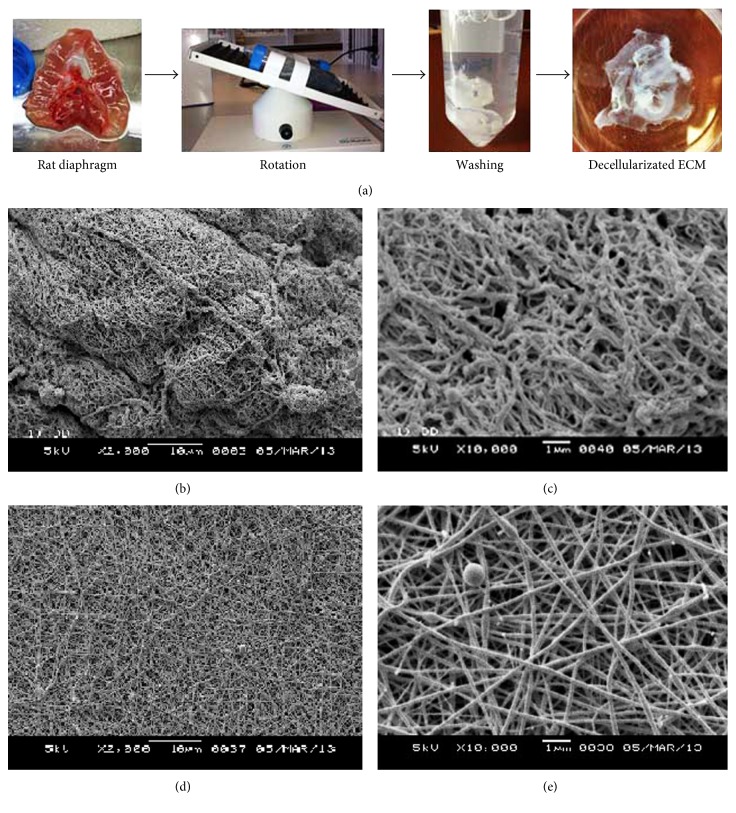
(a) A procedure of decellularization of rat diaphragm. (b, c) Results of TEM scan for the normal rat diaphragms. (d, e) Results of TEM scan for the decellularizated ECM scaffolds of rat diaphragms.

**Figure 6:**
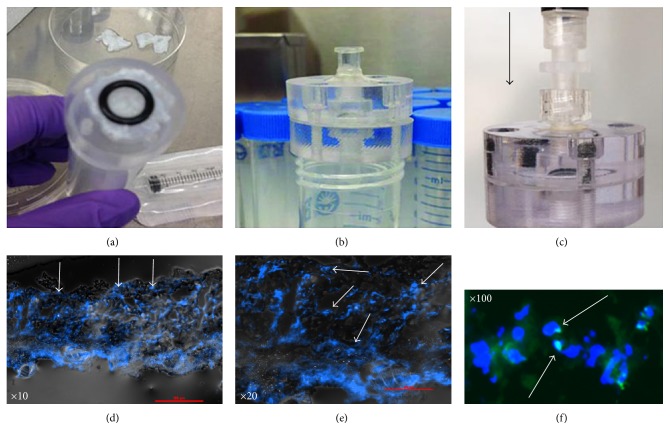
The process of cellular infusion in vitro. (a) A decellularized ECM scaffold is placed onto the top of an infusion chamber. (b) Position and fix ECM scaffold. (c) Begin infusing donor cells with culture medium into a decellularized ECM scaffold. (d, e) Histological analysis of cell distribution into scaffold by using DAPI staining. (f) Distribution test of fluorescent GFP-labeled donor cells in the ECM scaffold after completing infusion.

The ECM scaffold tissues were identified histologically by H&E or DAPI staining to verify the lack of surviving resident cells after decellularization (Figure 5). TEM examination for the ECM scaffolds also indicated that there were no changes in tissue structure or architecture before (Figures 5(a) and 5(b)) and after (Figures 5(c) and 5(d)) decellularization. We selected a suitable size of ECM scaffold to develop the composite tissue to allow diaphragmatic defect repair with donor hAFMSCs by using our infusion system in vitro (Figures 6(a)–6(c)).

The ECM scaffold was spread across one of the polycarbonate pieces and covered with the O-ring as shown in Figure 6(a). The second polycarbonate phalange art was placed on top of the O-ring and scaffold. The clamping screws were uniformly tightened to compress the scaffold and O-ring against the polycarbonate pieces to create a fluid-tight seal. A 5 mL syringe was loaded with a volume of donor cells, and a T luer fitting was affixed to the end of the syringe. An analog pressure gauge was attached to the T fitting, with the third end inserted into the infusion device. A syringe was attached to the device without the T fitting and pressure gauge (Figure 6(c)). A constant hydrostatic pressure was applied by manually pushing on the syringe and monitoring the pressure gauge. The pressure was maintained at constant levels for various durations. With histological analysis, we detected that the perfusion system could improve donor cell implantation into the scaffold, shown by DAPI staining (Figures 6(d) and 6(e)) and the tracking of the GFP-labeled cells (Figure 6(f)).

### 3.3. Stress/Strain Testing for Rat Diaphragm or Decellularized ECM Scaffolds

**Figure 7:**
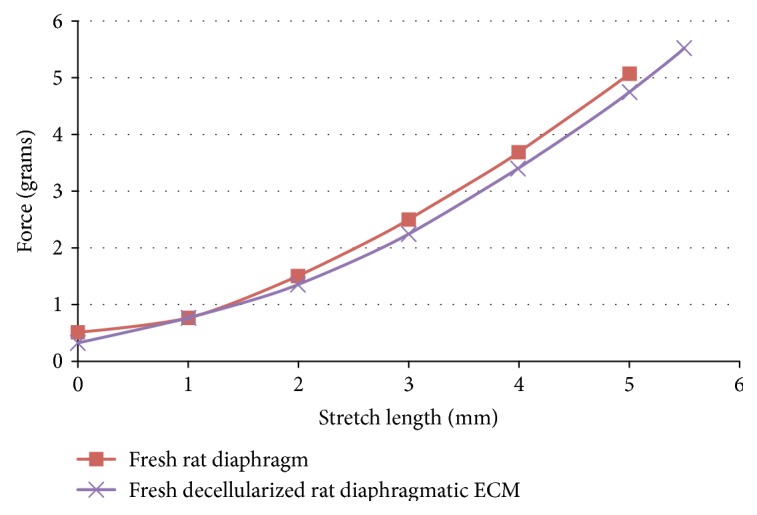
Modeling testing for the decellularized ECM scaffolds in vitro. Within the maximum stretch distance of 6.0 mm and maximum force of 6.0 grams applied to the ECM scaffolds, the results showed the similarity of stress/strain between the fresh rat diaphragms and the decellularized ECM scaffolds of rat diaphragms.

Figure 7 shows that the decellularized ECM of rat diaphragms developed a tension (> 90%) similar to that freshly isolated rat diaphragms.

### 3.4. Physiological Recovery after Natural Repair of the Diaphragmatic Defect

We tested the contractile force generation of all patch repaired tissues and contralateral control diaphragmatic tissues. Tissue contractile testing by single pulse (maximum force) stimulation and testing of modular strength, for example, train pulses (T-P) stimulation, were recorded separately. Single pulse stimulation of explanted hemidiaphragms repaired with the composite patch generated 77 ± 8% of the contralateral hemidiaphragm force compared to 50 ± 8% with the decellularized ECM scaffolds of rat diaphragms patch alone (*p* = 0.03). However, the composite patch hemidiaphragms had closer to normal (1.0 MPa) modular tensile strength at 1.2 ± 0.0 MPa than hemidiaphragms repaired with the decellularized ECM scaffolds patch at 1.4 ± 0.1 MPa (*p* = 0.05) (Table 1).

### 3.5. Histological Analyses of Healing Diaphragms

**Figure 8:**
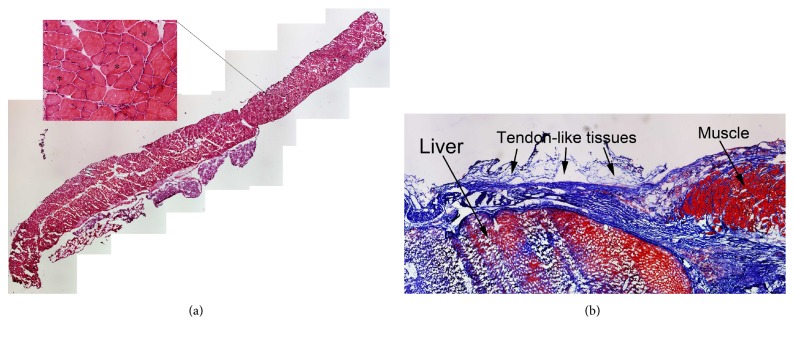
H&E staining to identify the muscular-tendon regrowth in the rat diaphragmatic defect. (a) Regenerated muscle tissue within the ECM scaffold after repairing diaphragmatic defect four months later. (b) Tendon-like tissue (arrows, blue tissue) can be detected connecting new muscle tissue within the repaired rat diaphragm. ∗ indicates central nuclei of regenerative muscle fibers; arrows indicates tendon-like tissue.

**Figure 9:**
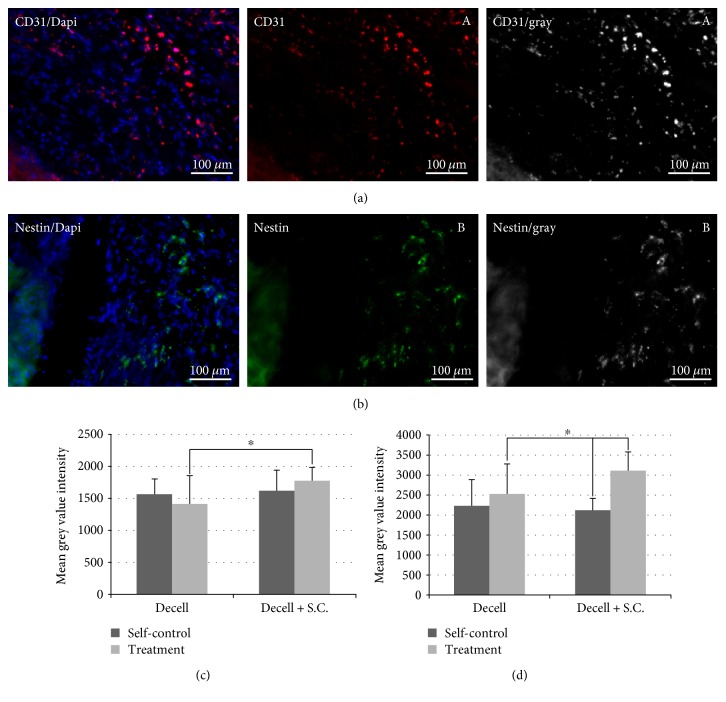
Immunohistochemical analysis of the regrowth tissues within the rat diaphragm. (a) CD31 positive cells (red) represent the vascularity that can be detected within the repaired rat diaphragmatic tissue. (b) Nerve regeneration can be evaluated by Nestin staining (green) within the implanted ECM scaffolds. We also measured the expression level of CD31 (A) and Nestin (B) through quantities of their gray fluorescence. The measurements indicated the revascularization (c) and reinnervation (d) that occurred in the repaired defect. ^∗^*p* < 0.05.

The repaired diaphragm was composed of filled and regenerated muscle tissues (center nuclei myofibers, Figure 8(a)), and a segment of repaired diaphragm demonstrated tendon-like tissues (trichrome staining blue colors, Figure 8(b)). These regenerated tissues were built with supporting decellularized ECM scaffolds. With immunostaining, we have discovered that the new diaphragm tissues were developed with innervation (ingrowth peripheral nerves) and vascularization (Figures 9(a) and 9(b)) four months after tissue repair. These immunohistochemical stains were analyzed by using ImageJ software, measuring the minimum, maximum, and the mean grey values of the threshold area (Figures 9(a) and 9(b)). Decellularized ECM tissue with cell compositions resulted in the higher number of densities in both vascular (CD31, Figure 9(a)) and neuronal (Nestin, Figure 9(b)) positive cells, indicating accelerated cellular infiltration and regeneration compared to the group of decellularized ECM scaffolds alone.

## 4. Discussion

It has been suggested that the combination of biomaterial scaffolds with stem cells can accelerate tissue regeneration and result in tissue repair with natural structural and functional properties [38, 39]. However, challenges remain in distributing cells into 3D scaffolds during the tissue engineering processes [36, 40]. Several methods have been tested for seeding stem cells into scaffolds, such as the use of 3D printers, growth factors, or chemical stimulation, and coculture scaffolds with stem cells in vitro [35, 41–43]. In this study, we used a cell infusion system that could successfully deliver donor cells into a 3D ECM scaffold, to build a composite tissue in vitro. Though, the decellularized ECM scaffold alone can be used to repair the tissue defect in rat diaphragms, the composite tissues that combined scaffold and hAFMSC improved the healing of the diaphragmatic defects physiologically and histologically. Similar studies of skeletal muscle or heart tissue repair using engineered tissues have indicated that the composite tissues with stem cells could accelerate healing processes and improve functional tissue recovery [21, 36, 44–46]. Additionally, the benefit of hAFMSCs is autologous cell sourcing to minimize immune responses. Further, there is an ample time window of ~2 months to allow expansion of the donor hAFMSCs for use at the time of birth.

We measured the diaphragmatic function using electrical stimulation ex vivo. These innovative results reflect the recovery of muscle, tendon, neuron-muscular-junction, and muscle-tendon-junction within the engineered tissues. Moreover, the result of electrical stimulation was confirmed by histological analysis. In physiological terms, the composite patches demonstrated maximum contractile force and tensile properties closer to native tissues as compared to decellularized ECM scaffolds alone. Histological results also indicated that the use of decellularized ECM scaffolds alone or composite tissues could repair the defects with different levels of revascularization. We noted a difference in Nestin staining between decellularized ECM scaffolds and the composite tissues, potentially representing never ingrowth. Although there was no mechanical failure or reherniation, there is potential for these scaffolds to be mechanically inadequate when scaling up to models such as pigs or humans. We are currently investigating the use of decellularized ECM scaffolds seeded with hAFMSCs, combined with other matrices, such as natural silks or biomaterials to improve the initial tensile properties following implantation and scaling up to porcine models. Additionally, improved seeding methods may be required for larger patches, possibly via a sandwich (ECM-cells-ECM) composite tissue. The strategy to use more than one layer of decellularized ECM scaffolds of diaphragms to potentially support the repair of large diaphragmatic defects or emergency uses is clinically relevant.

## 5. Conclusion

The native decellularized ECM scaffold from rat diaphragms can be used as a support material for repairing rat diaphragmatic defects. However, the composite biological patches that combine decellularized ECM scaffolds with human amniotic fluid-derived multipotent stromal cells (hAFMSCs) could improve diaphragmatic biomechanical function and modular tensile strength. The repair with composite tissues is also associated with improved innervation, vasculogenesis, and muscular-tendon regrowth.

## Figures and Tables

**Table 1:** 

	Decell ± SEM (*n*)	Decell + hAFMSCs ± SEM (*n*)	*p* value
Max force (% native)	50 ± 8% (9)	77 ± 8% (7)	0.03
Modular strength (P-T, MPa)	1.4 ± 0.1 (13)	1.2 ± 0.0 (11)	0.05
